# Supplementary material for: Personalized whole-brain neural mass models reveal combined Aβ and tau hyperexcitable influences in Alzheimer’s disease
Source: Commun Biol. 2024 May 4;7:528. doi: 10.1038/s42003-024-06217-2 (PMC11069569; doi:10.1038/s42003-024-06217-2)
Supplement: Supplementary file 2 — Supplementary Information [file 42003_2024_6217_MOESM2_ESM.pdf]

Supplementary Information

Personalized whole-brain neural mass models reveal combined Aβ and tau hyperexcitable influences in Alzheimer's disease

Lazaro M. Sanchez-Rodriguez[1,2,3], Gleb Bezgin[1,2,3,4], Felix Carbonell[5], Joseph Therriault[1,2,4], Jaime Fernandez-Arias[1,2,4], Stijn Servaes[1,2,4], Nesrine Rahmouni[1,2,4], Cecile Tissot[1,2,4], Jenna Stevenson[1,2,4], Thomas K. Karikari[6,7], Nicholas J. Ashton[6,8,9,10], Andréa L. Benedet[6], Henrik Zetterberg[6,11,12,13,14,15], Kaj Blennow[6,15], Gallen Triana-Baltzer[16], Hartmuth C. Kolb[16], Pedro Rosa-Neto [1,4], Yasser Iturria-Medina[1,2,3*]

[1]Department of Neurology and Neurosurgery, McGill University, Montreal, Canada.
[2]McConnell Brain Imaging Centre, Montreal Neurological Institute, Montreal, Canada.
[3]Ludmer Centre for Neuroinformatics & Mental Health, Montreal, Canada.
[4]McGill University Research Centre for Studies in Aging, Douglas Research Centre, Montreal, Canada.
[5]Biospective Inc., Montreal, Canada.
[6]Department of Psychiatry and Neurochemistry, Institute of Neuroscience and Physiology, The Sahlgrenska Academy at the University of Gothenburg, Mölndal, Sweden.
[7]Department of Psychiatry, School of Medicine, University of Pittsburgh, Pittsburgh, PA, USA.
[8]King's College London, Institute of Psychiatry, Psychology and Neuroscience Maurice Wohl Institute Clinical Neuroscience Institute London UK.
[9]NIHR Biomedical Research Centre for Mental Health and Biomedical Research Unit for Dementia at South London and Maudsley NHS Foundation London UK.
[10]Centre for Age-Related Medicine, Stavanger University Hospital, Stavanger, Norway.
[11]Department of Neurodegenerative Disease, UCL Institute of Neurology, Queen Square, London, UK.
[12]UK Dementia Research Institute at UCL, London, UK.
[13]Hong Kong Center for Neurodegenerative Diseases, Clear Water Bay, Hong Kong, China.
[14]Wisconsin Alzheimer's Disease Research Center, University of Wisconsin School of Medicine and Public Health, University of Wisconsin-Madison, Madison, WI, USA.
[15]Clinical Neurochemistry Laboratory, Sahlgrenska University Hospital, Mölndal.
[16]Neuroscience Biomarkers, Janssen Research & Development, La Jolla, California, USA.

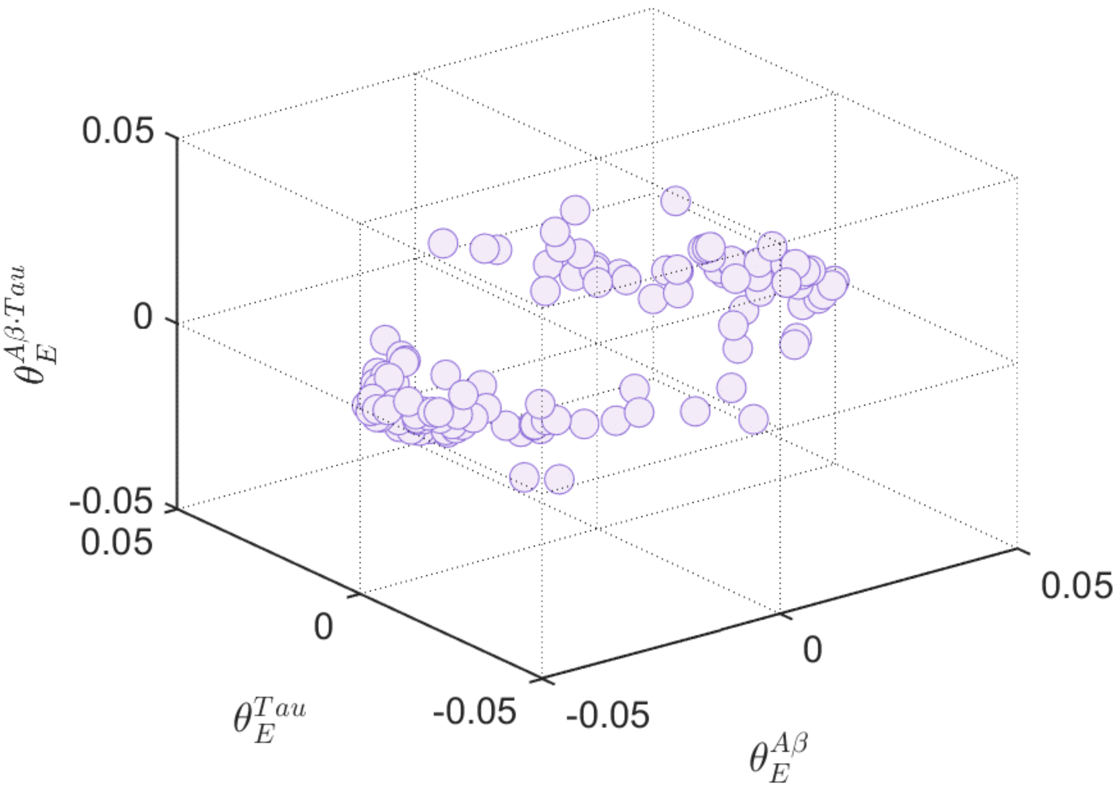

Supplementary Figure 1. Estimated neuronal activity influences by *Aβ, tau and Aβ·tau*. Each dot represents a subject-specific set of pathophysiological effects on neuronal excitability, obtained through parameter estimation following the model of equation (1) in the main text. The axes correspond to the Aβ, tau and Aβ·tau pathophysiological influence weights, respectively.

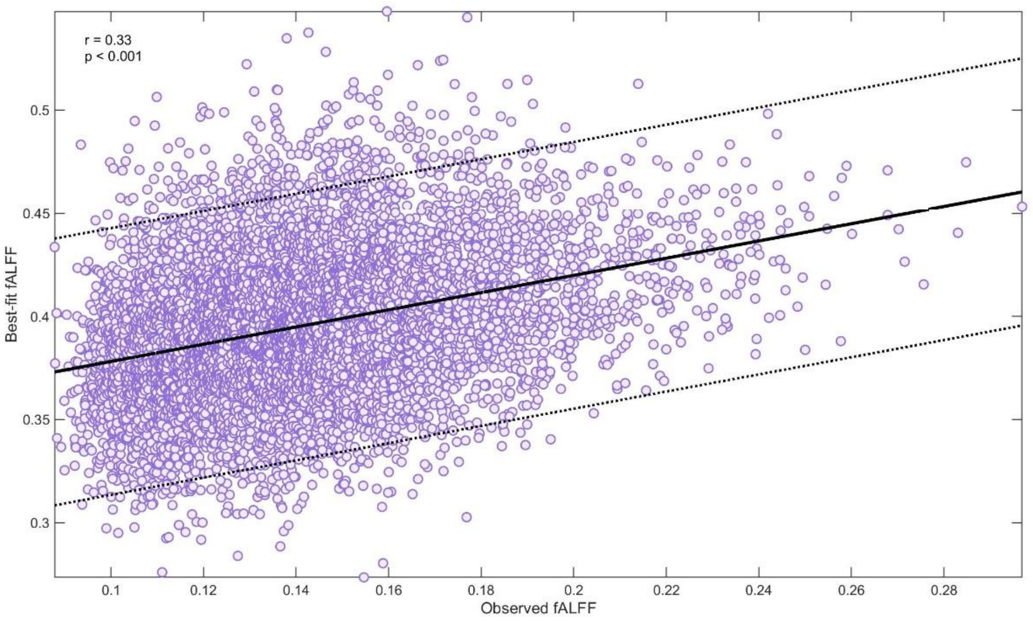

Supplementary Figure 2. Best-fit model fALFF vs observed fALFF. Each data point corresponds to a brain region in the parcellation and subject in the cohort. The results of a Pearson correlation analysis are inserted in the bottom right. The error bands denote 95% confidence intervals.

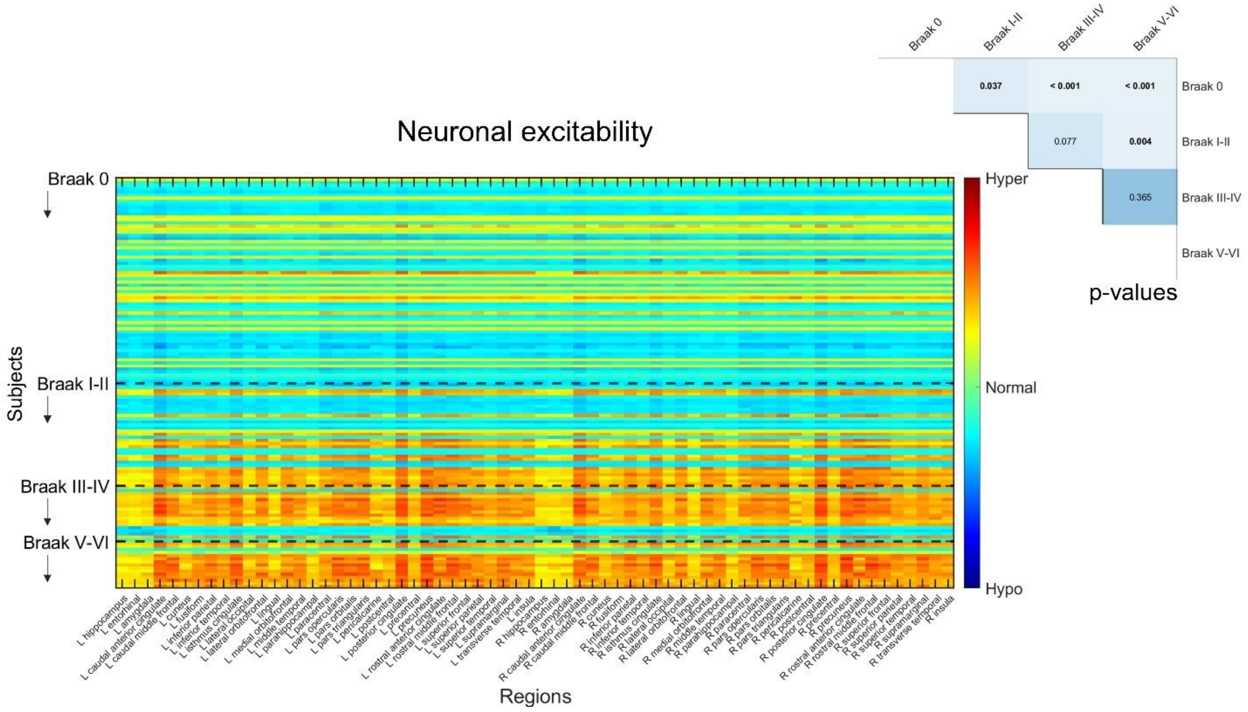

Supplementary Figure 3. Neuronal excitabilities under the influence of *Aβ, tau and Aβ·tau*. Participants were grouped according to Braak stages in this figure, to understand tau's contribution to the individually estimated biological profiles. Inferred neuronal excitability values for the brain regions of interest ("y"-axis) and all subjects ("x"-axis). Within a group, subjects appear according to their existing ordering in the anonymized database. Warm colors represent hyperexcitability of the region in the subject's brain and cool colors denote hypoexcitable states. Results of ANCOVA post-hoc t-tests for the above-mentioned groups, with the average intra-brain excitability values as response variable and age and sex as covariates appear in the upper right corner. P-values in bold fonts represent differences at a 5% significance level or lower. This figure is analogous to Figure 3.

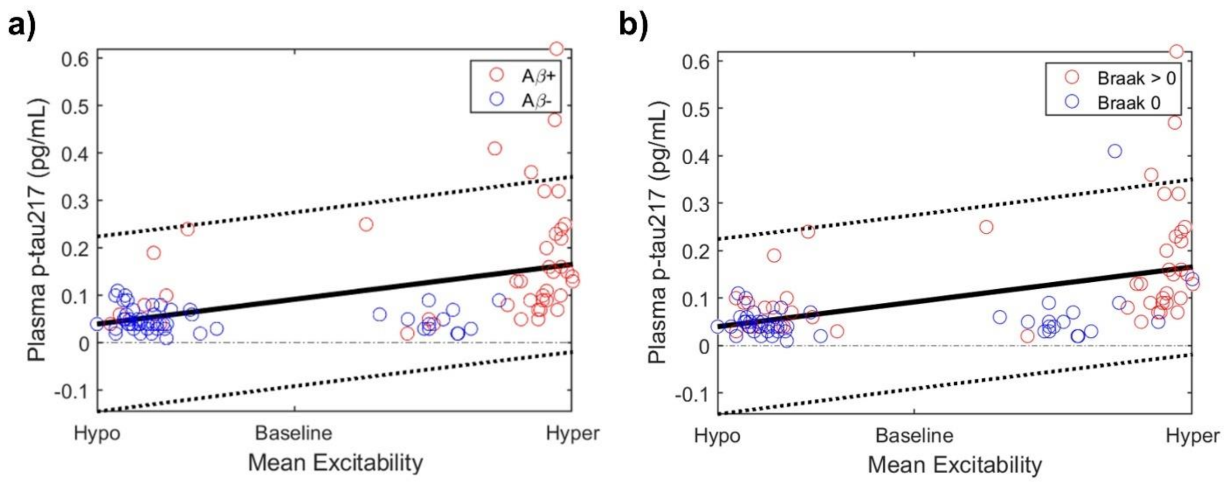

Supplementary Fig 4. Amyloid-beta and tau tangle status of the subjects in the relationship plasma biomarkers vs estimated neuronal excitability. a) Aβ status. Subjects are highlighted in different colors according to Aβ-positivity. **b**) Tau Braak stages. Subjects are highlighted in different colors based on whether they present significant tau involvement (Braak > 0) or not. This figure complements Figure 3, depicting the excitability distribution that occurs with disease states. Only panel c of Figure 4 has been detailed. Others are analogous.

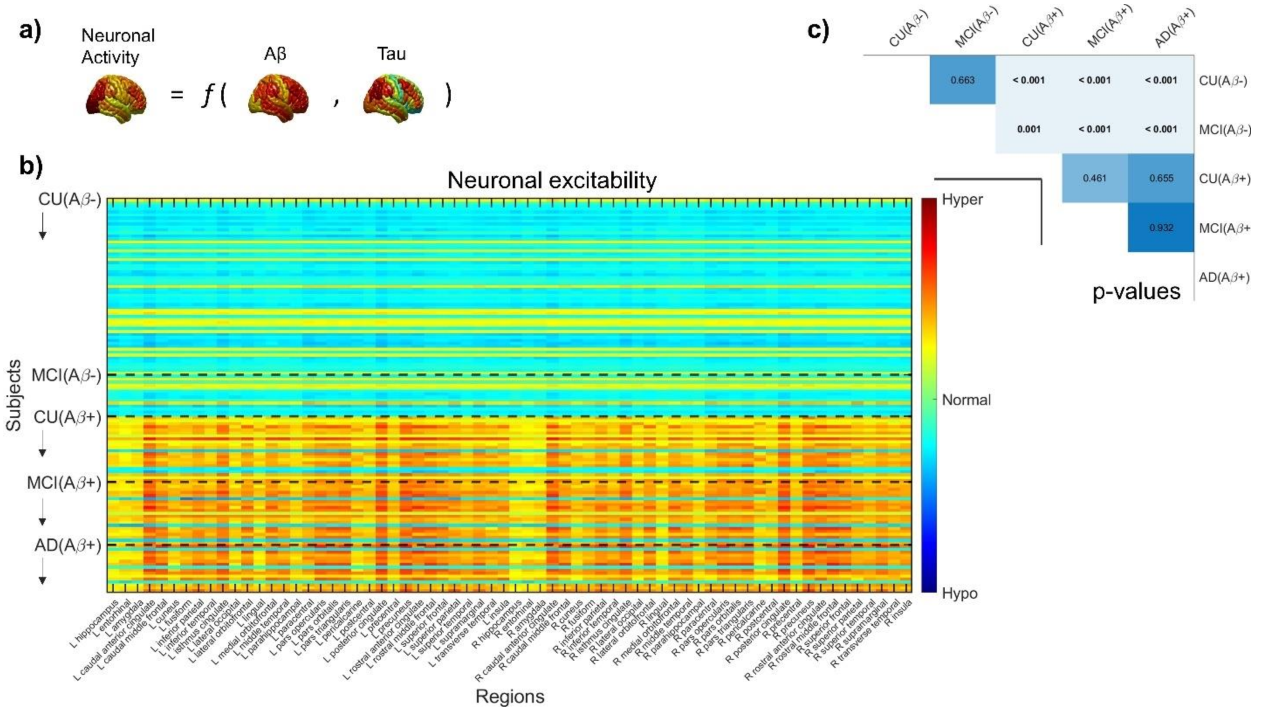

	CU(Aβ-)	MCI(Aβ-)	CU(Aβ+)	MCI(Aβ+)	AD(Aβ+)
CU(Aβ-)		0.663	**< 0.001**	**< 0.001**	**< 0.001**
MCI(Aβ-)			**0.001**	**< 0.001**	**< 0.001**
CU(Aβ+)				0.461	0.655
MCI(Aβ+					0.932
AD(Aβ+)					

Supplementary Figure 5. Neuronal excitabilities under the influence of *Aβ and tau* (participants grouped according to Aβ-positivity). **a**) Schematic representation of the influence model. **b**) Inferred neuronal excitability values for the brain regions of interest ("y"-axis) and all subjects ("x"-axis). Within a group, subjects appear according to their existing ordering in the anonymized database. Warm colors represent hyperexcitability of the region in the subject's brain and cool colors denote hypoexcitable states. **c**) Results of ANCOVA post-hoc t-tests for the above-mentioned groups, with the average intra-brain excitability values as response variable and age and sex as covariates. P-values in bold fonts represent differences at a 5% significance level or lower.

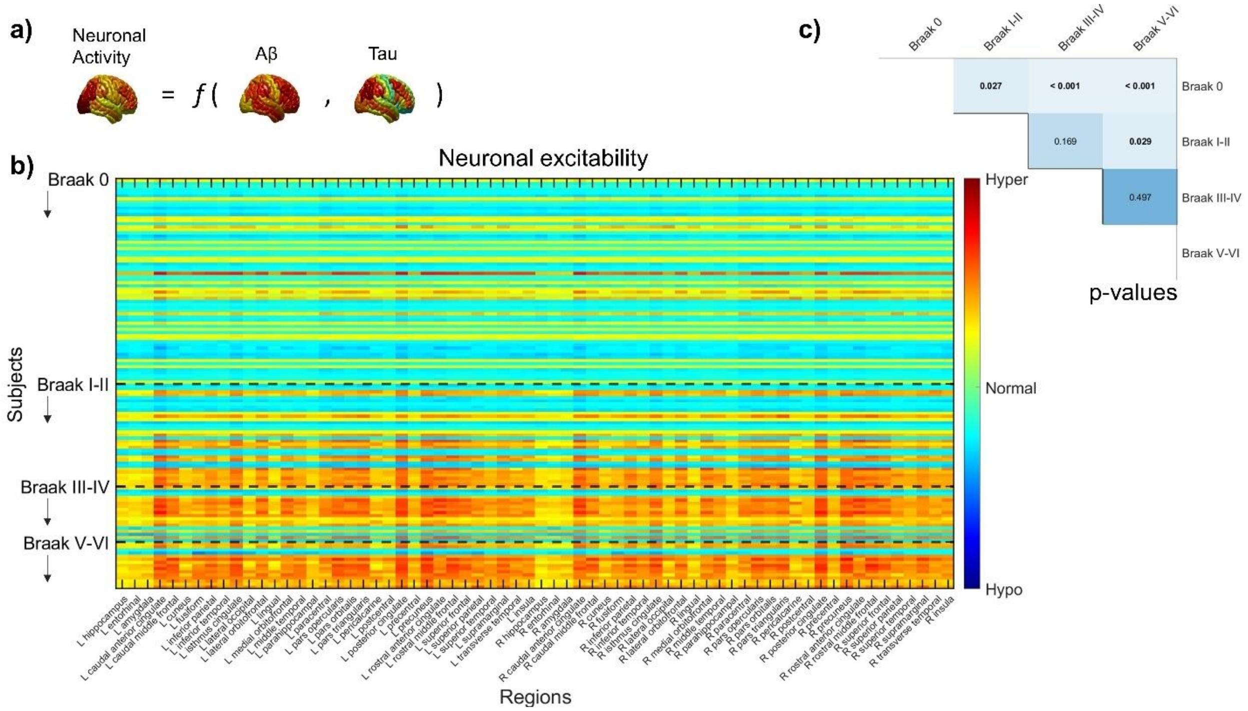

Supplementary Figure 6. Neuronal excitabilities under the influence of *Aβ and tau* (participants grouped according to Braak stages). a) Schematic representation of the influence model. **b**) Inferred neuronal excitability values for the brain regions of interest (“y”-axis) and all subjects (“x”-axis). Within a group, subjects appear according to their existing ordering in the anonymized database. Warm colors represent hyperexcitability of the region in the subject’s brain and cool colors denote hypoexcitable states. **c**) Results of ANCOVA post-hoc t-tests for the above-mentioned groups, with the average intra-brain excitability values as response variable and age and sex as covariates. P-values in bold fonts represent differences at a 5% significance level or lower.

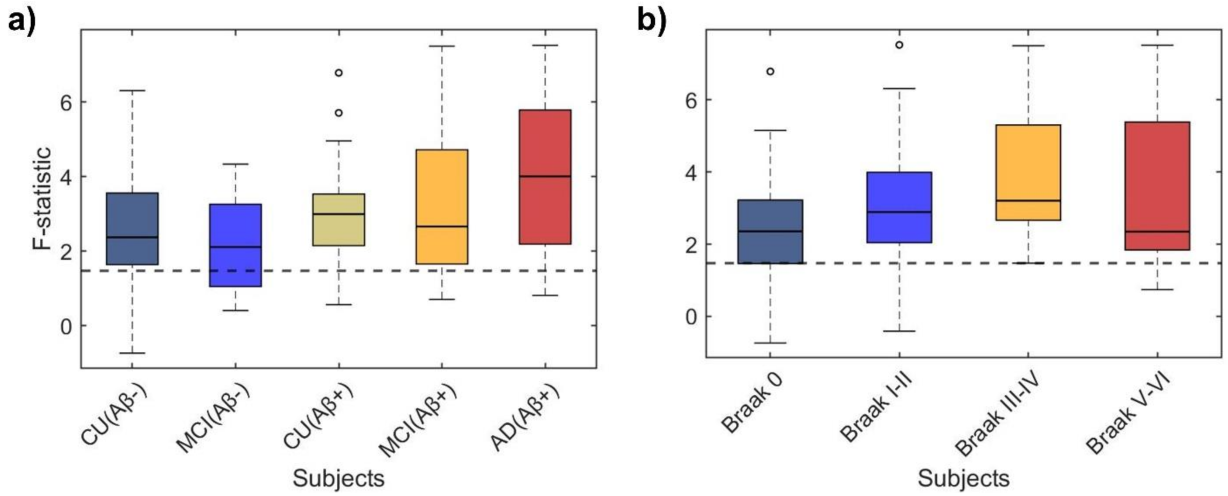

Supplementary Figure 7. Contribution of Aβ and tau accumulations to explain the observed neuronal activity alterations. Participants were separated into groups according to their clinical diagnosis (CU, MCI, AD) and Aβ-positivity (**a**) or in-vivo Braak staging (**b**). Statistical comparison was established through subject-wise *F*-tests between models with and without Aβ, tau and Aβ·tau maps (4 and 1 parameters, respectively). The critical threshold (black dashed lines) corresponds to a statistically significant ($p < 0.05$) improvement due to Aβ, tau and Aβ·tau influences on neuronal excitability, accounting for the increase in adjustable model parameters.

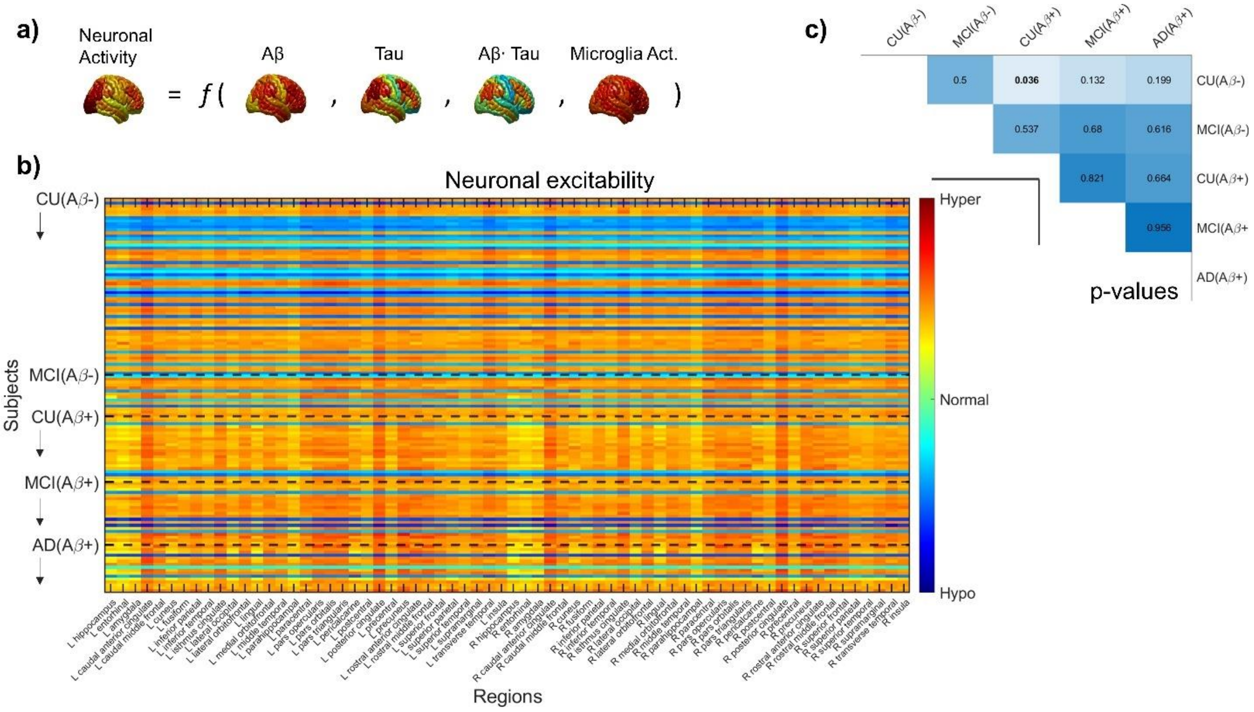

Supplementary Figure 8. Neuronal excitabilities under the influence of *Aβ, tau, Aβ·tau and microglial activation* (participants grouped according to Aβ-positivity). **a**) Schematic representation of the influence model. **b**) Inferred neuronal excitability values for the brain regions of interest ("y"-axis) and all subjects ("x"-axis). Within a group, subjects appear according to their existing ordering in the anonymized database. Warm colors represent hyperexcitability of the region in the subject's brain and cool colors denote hypoexcitable states. **c**) Results of ANCOVA post-hoc t-tests for the above-mentioned groups, with the average intra-brain excitability values as response variable and age and sex as covariates. No significant differences between groups were observed.

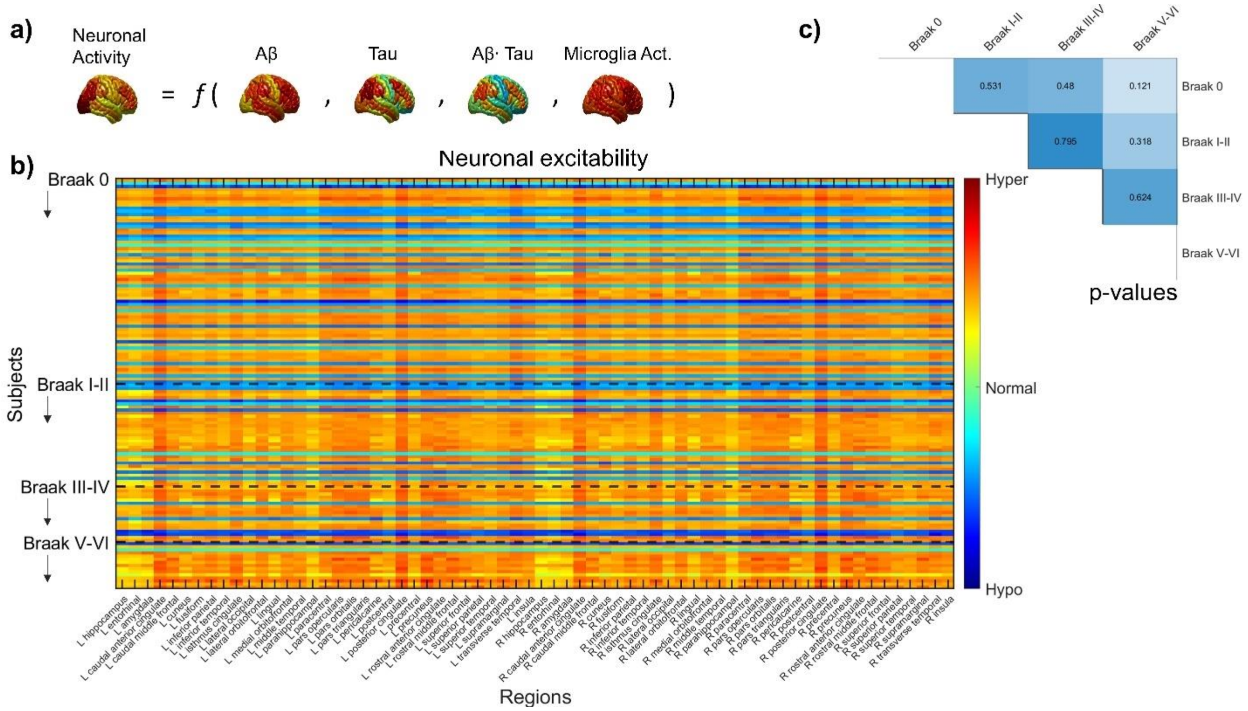

Supplementary Figure 9. Neuronal excitabilities under the influence of *Aβ, tau, Aβ·tau and microglial activation* (participants grouped according to Braak stages). a) Schematic representation of the influence model. **b**) Inferred neuronal excitability values for the brain regions of interest (“y”-axis) and all subjects (“x”-axis). Within a group, subjects appear according to their existing ordering in the anonymized database. Warm colors represent hyperexcitability of the region in the subject’s brain and cool colors denote hypoexcitable states. **c**) Results of ANCOVA post-hoc t-tests for the above-mentioned groups, with the average intra-brain excitability values as response variable and age and sex as covariates. P-values in bold fonts represent differences at a 5% significance level or lower.

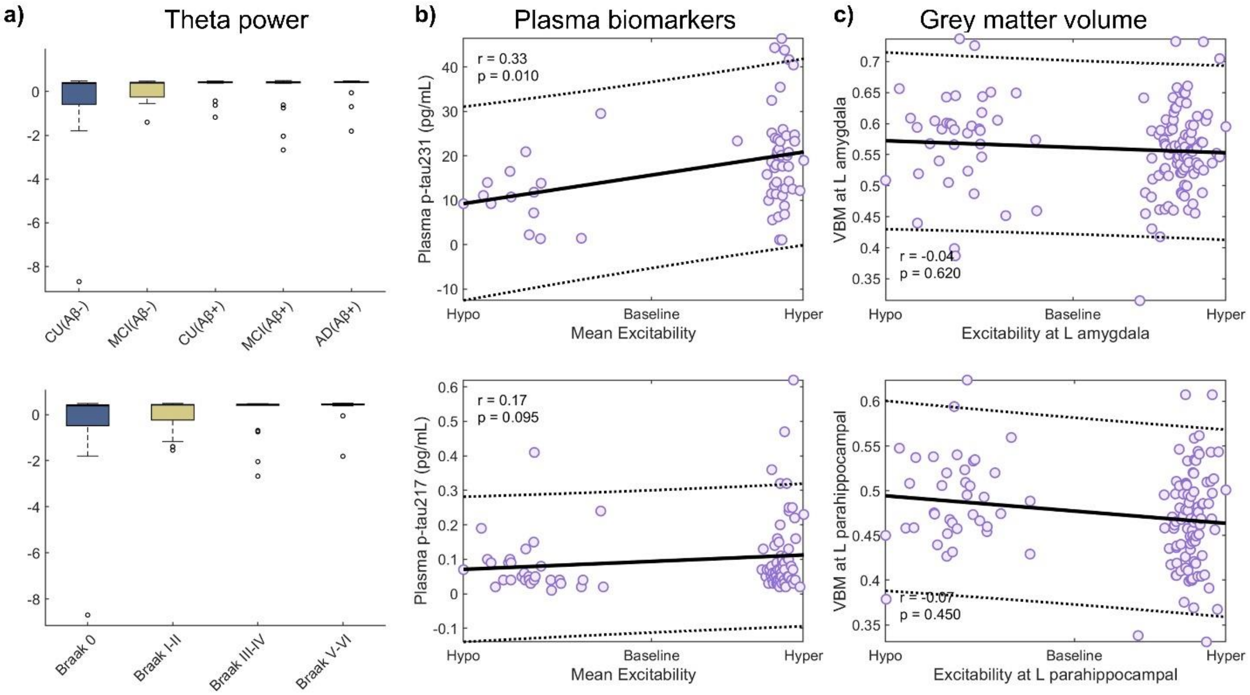

Supplementary Figure 10. Biophysical context of the *Aβ, tau, Aβ·tau and microglial activation* model's results. a) Ratio of power in the theta band (4–8 Hz) of the regional excitatory input currents (the E/MEG is proportional to the excitatory input current). No significant differences were observed in ANCOVA post-hoc t-tests for the clinical groups. The ratio of power in the alpha1 band and mean excitatory firings behave analogously. See Figure 2 for more details. **b**) Spearman's correlation analyses for the associations between the participants' estimated average intra-brain excitabilities and the plasma biomarkers p-tau231 and p-tau217. Additionally, no significant relationship existed with p-tau181 and a decreased correlation (compared to the results reported in Figure 4) was observed for GFAP ($r = 0.20$, $p = 0.034$). **c**) Spearman's correlation analyses for the associations between the participants' estimated local excitability values at the left amygdala and left parahippocampal gyrus and the regional grey matter volumes. Compare to Figure 4f-g. In general, the significant relationships with grey matter volumetric AD reductions in the model not considering microglial activation (Figure 4 and Supplementary Table 3) were not observed. Weak associations ($r < 0.23$ in all cases) existed for regions outside the medial temporal lobe.

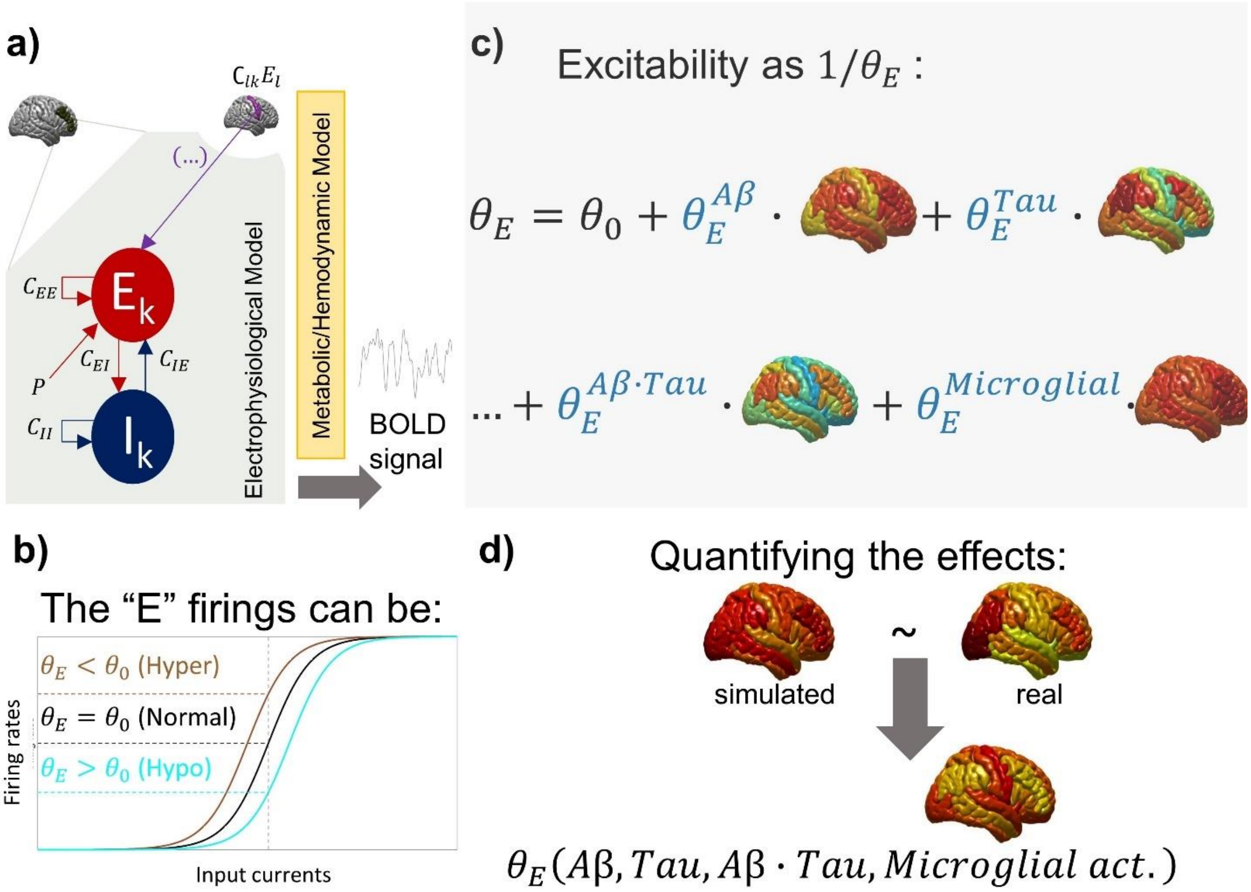

Supplementary Figure 11. Overview of integrating pathophysiological information into the computational brain activity decoder for Alzheimer's disease. a) Each brain region, k, is modeled as coupled excitatory and inhibitory populations. For example, C_{EI} represents the strength of the excitatory connection with the inhibitory neural mass. Additionally, the excitatory population receives an unspecific local stimulus that accounts for unmodeled interactions, P, and excitatory inputs from other regions via a connectivity matrix obtained from diffusion MRI (C_{lk}). The excitatory and inhibitory inputs feed a metabolic/hemodynamic model that simulates the resting-state BOLD signal (fMRI) for the given region. **b**) In the study, the excitatory firing rates are obtained by using sigmoid functions with varying firing thresholds. A low sigmoidal threshold value means high firing rates ("hyperexcitable state") at a given input current, while the opposite is termed "hypoexcitability", as compared to baseline firing conditions. **c**) The subject-specific influence of pathophysiological factors on neuronal activity is modeled as linear changes on a region's firing threshold due to the competition/contribution of the regional pathological loads. In Alzheimer's disease, we assume that the threshold can be, in general, affected by Aβ plaques, tau tangles, the interaction of Aβ and tau (modeled as the regional multiplication of the participant's Aβ and tau SUVRs), and microglial activation. Thus, the anatomical representations in the figure gather PET SUVRs corresponding to these factors in 66 regions of interest. The proportionality constants (θ_E^{Ab}, θ_E^{Tau}, $\theta_E^{Ab \cdot Tau}$, $\theta_E^{Microglial\ act.}$) characterize the subject-specific global influence of the pathologies. **d**) We infer these pathological influences by maximizing the similarity between the observed neuronal activity biomarkers (i.e., the fractional amplitude of low-frequency

fluctuations in the regional BOLD signals) and the analogous indicators in the simulated signals, for each of the participants. With the obtained global influences, we reconstruct hidden excitability maps based on the pathology-dependent neuronal firing thresholds.

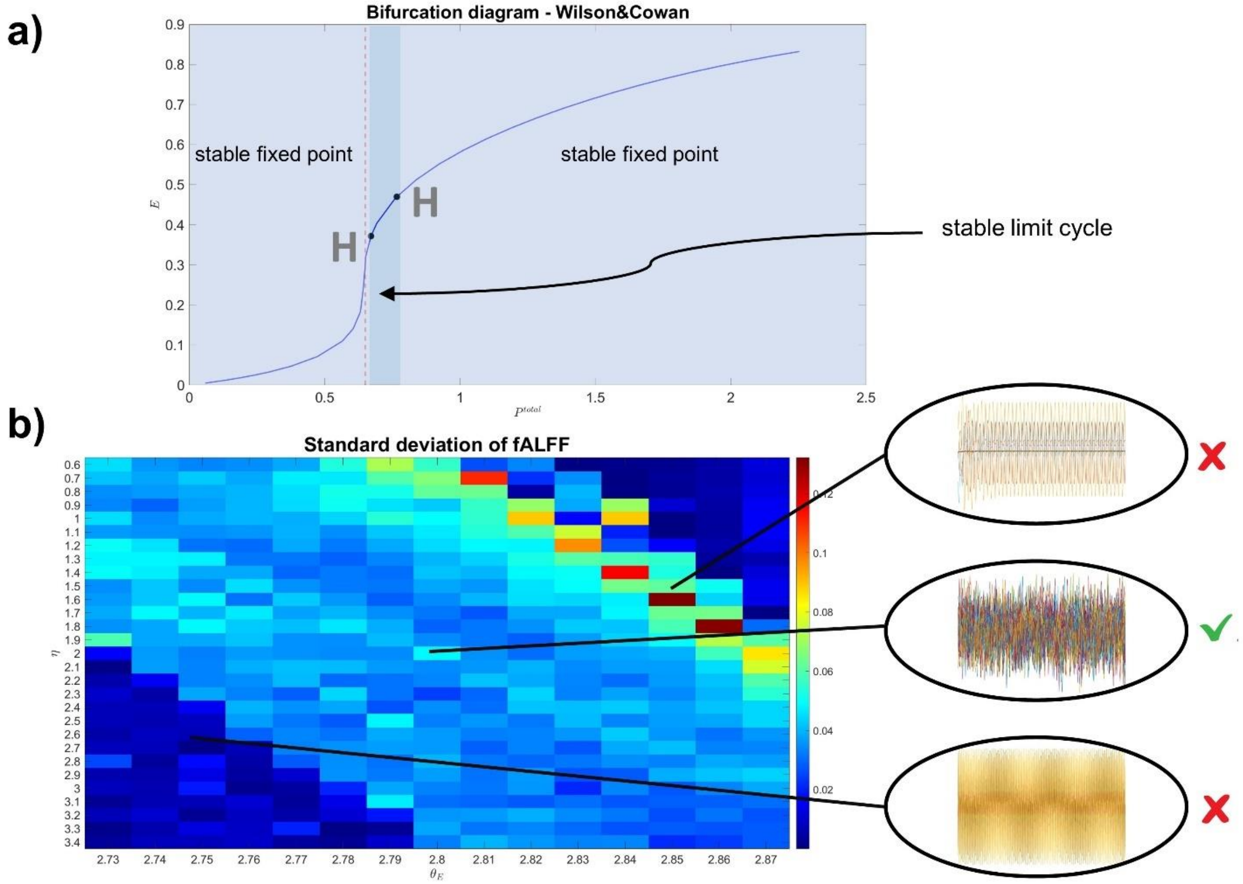

Supplementary Figure 12. Selecting connectivity parameters for the neuronal activity models. a) Bifurcation diagram of an isolated Wilson-Cowan module with external input, P^{total}, as bifurcation parameter and the resting parameters as in Supplementary Table 6. Supercritical Hopf bifurcations appear at the points marked as "H". Within the region given by these two points, and for all biologically plausible values of the state variables (shown is the excitatory firing rate, E), only one stable state exists: limit cycle oscillations. In all the calculations, the constant external input received by the excitatory population, P, was thus set to a value just under the first Hopf bifurcation ($P = 0.65$, red dashed line). By selecting such a working point, we let the cortico-cortical inputs (given by $\frac{\eta}{N}\sum_{l=1,l\neq k}^{N} C_{lk}E_l$, *Methods, Integrative neuronal activity simulator*) drive the fully connected electrophysiological system towards the sustained oscillations solution. **b)** Empirical analysis of the BOLD signals simulated through metabolic/hemodynamic transformations of the excitatory and inhibitory firings (see also Supplementary Table 7). Shown are the standard deviations of the regional fractional amplitude of low-frequency fluctuations (fALFF) for several values of the global coupling strength (η) scaling the anatomical connectivity matrix and the total threshold for activation of the excitatory sigmoidal firing function (θ_E). For some combinations of η and θ_E, the set of BOLD signals returned by the model are highly uniform and coupled, thus not realistic (red crosses on the right). We selected a working value of $\eta = 2$. The threshold parameter, θ_E, which determines excitability in the model, can then exist in a large and symmetrical interval ($\theta_E \in [2.75,2.85]$) that produces plausible BOLD signals.

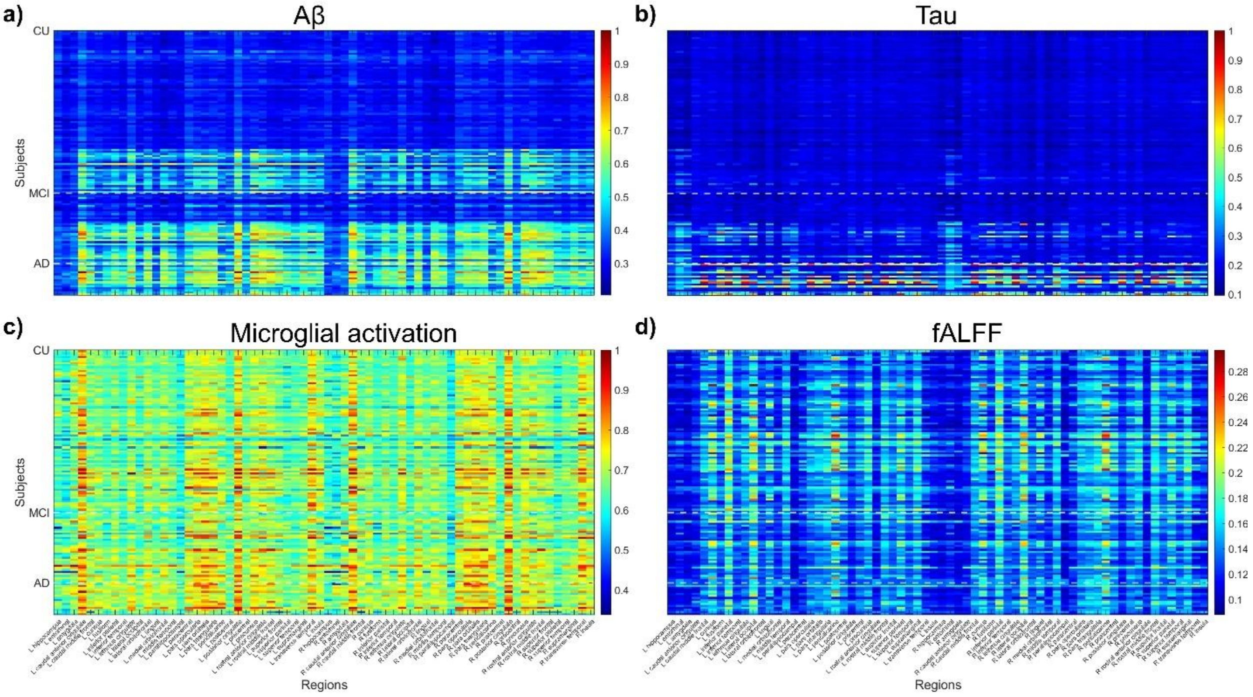

Supplementary Figure 13. Distributions of the processed data that was utilized in the study. **a**) Aβ PET SUVRs **b**) Tau PET SUVRs **c**) Microglial activation PET SUVRs **d**) Fractional amplitudes of low-frequency fluctuations (fALFF) from fMRI. Subjects are organized by clinical groups (CU, MCI, AD) and appear in the same order as in Figure 3 within a given clinical group. The SUVRs were normalized to the [0,1] interval by dividing by the absolute maximum value corresponding to each imaging modality.

Supplementary Table 1. Demographics of the samples

	CU	MCI	p	AD	p
Number of individuals (N)	81	35	-	16	-
Age (yrs), mean (s.d.)	71.15 (7.73)	72.17 (7.84)	0.517	69.45 (9.10)	0.438
Female, N (%)	63 (77.8)	17 (48.6)	0.004	8 (50.0)	0.031
Education (yrs), mean (s.d.)	15.26 (3.54)	15.63 (3.01)	0.591	14.31 (3.38)	0.327
APOE ε4 carriers, N (%)	24 (29.6)	17 (48.6)	0.059	7 (43.8)	0.379
MMSE, mean (s.d.)	29.28 (0.97)	28.40 (1.54)	< 0.001	20.25 (7.20)	< 0.001
Aβ+, N (%)	20 (24.7)	23 (65.7)	0.006	16 (100.0)	< 0.001

The reported p-values are for comparisons to cognitively unimpaired (CU) subjects. P-values for age, education and MMSE indicate values assessed with two-sided independent-samples t-tests. For the resting variables (sex, *APOE* ε4 status and Aβ+), Fischer exact test were performed. CU cognitively unimpaired; MCI mild cognitive impairment; AD Alzheimer's disease; *APOE* ε4, apolipoprotein epsilon 4; MMSE, Mini-Mental State examination.

Supplementary Table 2. Summary demographics of the groups that were compiled for post-hoc analyses of the individually estimated pathophysiological quantities

	CU(Aβ-)	MCI(Aβ-)	CU(Aβ+)	MCI(Aβ+)	AD(Aβ+)
Number of individuals (N)	59	14	22	21	16
Age (yrs), mean (s.d.)	70.04 (8.29)	72.89 (6.54)	74.11 (5.03)	71.68 (8.73)	69.45 (9.10)
Female, N (%)	45 (76.3)	6 (42.9)	18 (81.8)	11 (52.4)	8 (50.0)
Education (yrs), mean (s.d.)	15.59 (3.79)	14.86 (3.53)	14.36 (2.61)	16.14 (2.57)	14.31 (3.38)
APOE ε4 carriers, N (%)	13 (22.0)	2 (14.3)	11 (50.0)	15 (71.4)	7 (43.8)
MMSE, mean (s.d.)	29.28 (0.96)	28.64 (1.39)	29.27 (1.03)	28.24 (1.64)	20.25 (7.20)
	Braak 0	Braak I-II	Braak III-IV	Braak V-VI	
Number of individuals (N)	66	33	18	15	
Age (yrs), mean (s.d.)	70.28 (8.01)	72.89 (8.24)	74.68 (5.09)	67.44 (7.72)	
Female, N (%)	45 (68.2)	24 (72.7)	10 (55.6)	9 (60.0)	
Education (yrs), mean (s.d.)	15.27 (3.61)	15.03 (3.41)	15.72 (2.42)	15.00 (3.51)	
APOE ε4 carriers, N (%)	16 (24.2)	11 (33.3)	14 (77.8)	7 (46.7)	
MMSE, mean (s.d.)	29.31 (0.89)	28.24 (2.09)	27.83 (2.81)	21.47 (8.24)	

CU cognitively unimpaired; MCI mild cognitive impairment; AD Alzheimer's disease; *APOE* ε4, apolipoprotein epsilon 4; MMSE, Mini-Mental State examination.

Supplementary Table 3. Results of statistical tests

Quantity	Test	Group 1	Group 2	F-Statistic	dfe	p
Ratio of power in theta band^	ANCOVA post-hoc t-tests	CU(Aβ-)	CU(Aβ+)	41.88	77	< 0.001
		CU(Aβ-)	MCI(Aβ+)	54.63	76	< 0.001
		CU(Aβ-)	AD(Aβ+)	41.84	71	< 0.001
		MCI(Aβ-)	CU(Aβ+)	6.50	32	0.016
		MCI(Aβ-)	MCI(Aβ+)	21.28	31	< 0.001
		MCI(Aβ-)	AD(Aβ+)	17.70	26	< 0.001
		Braak 0	Braak III-IV	14.34	80	< 0.001
		Braak 0	Braak V-VI	33.60	77	< 0.001
		Braak I-II	Braak V-VI	8.23	44	0.006
Ratio of power in alpha1 band^	ANCOVA post-hoc t-tests	CU(Aβ-)	CU(Aβ+)	31.78	77	< 0.001
		CU(Aβ-)	MCI(Aβ+)	33.40	76	< 0.001
		CU(Aβ-)	AD(Aβ+)	20.12	71	< 0.001
		MCI(Aβ-)	MCI(Aβ+)	12.14	31	0.001
		MCI(Aβ-)	AD(Aβ+)	7.36	26	0.011
		Braak 0	Braak III-IV	5.96	80	0.017
		Braak 0	Braak V-VI	16.95	77	< 0.001
		Braak I-II	Braak V-VI	8.26	44	0.006
Mean excitatory activity^	ANCOVA post-hoc t-tests	CU(Aβ-)	CU(Aβ+)	49.80	77	< 0.001
		CU(Aβ-)	MCI(Aβ+)	59.34	76	< 0.001
		CU(Aβ-)	AD(Aβ+)	46.93	71	< 0.001
		MCI(Aβ-)	CU(Aβ+)	8.86	32	0.005
		MCI(Aβ-)	MCI(Aβ+)	21.25	31	< 0.001
		MCI(Aβ-)	AD(Aβ+)	18.22	26	< 0.001
		Braak 0	Braak III-IV	15.35	80	< 0.001
		Braak 0	Braak V-VI	38.38	77	< 0.001
		Braak I-II	Braak V-VI	8.70	44	0.005
Average intra-brain excitability^	ANCOVA post-hoc t-tests	CU(Aβ-)	CU(Aβ+)	55.54	77	< 0.001
		CU(Aβ-)	MCI(Aβ+)	66.06	76	< 0.001
		CU(Aβ-)	AD(Aβ+)	54.19	71	< 0.001
		MCI(Aβ-)	CU(Aβ+)	9.96	32	0.003
		MCI(Aβ-)	MCI(Aβ+)	22.65	31	< 0.001
		MCI(Aβ-)	AD(Aβ+)	19.78	26	< 0.001
		Braak 0	Braak I-II	4.48	95	0.037
		Braak 0	Braak III-IV	18.02	80	< 0.001
		Braak 0	Braak V-VI	44.35	77	< 0.001
		Braak I-II	Braak V-VI	8.97	44	0.004

Plasma biomarkers	Spearman correlation	Mean excitability	p-tau181 (*r=0.39*)	NA	NA	0.003
		Mean excitability	p-tau231 (*r=0.38*)	NA	NA	0.003
		Mean excitability	p-tau217 (*r=0.52*)	NA	NA	< 0.001
		Mean excitability	GFAP (*r=0.32*)	NA	NA	< 0.001
Grey matter atrophy	Spearman correlation	Mean excitability	Mean VBM (*r=-0.23*)	NA	NA	0.009
		Excitability L_hippocampus	VBM L_hippocampus (*r=-0.20*)	NA	NA	0.024
		Excitability L_entorhinal	VBM L_entorhinal (*r=-0.26*)	NA	NA	0.024
		Excitability L_amygdala	VBM L_amygdala (*r=-0.33*)	NA	NA	< 0.001
		Excitability L_fusiform	VBM L_fusiform (*r=-0.17*)	NA	NA	0.045
		Excitability L_inf_temp	VBM L_inf_temp (*r=-0.23*)	NA	NA	0.009
		Excitability L_mid_temp	VBM L_mid_temp (*r=-0.22*)	NA	NA	0.013
		Excitability L_parahippoc	VBM L_parahippoc (*r=-0.25*)	NA	NA	0.004
		Excitability L_post_cing	VBM L_post_cing (*r=-0.20*)	NA	NA	0.020
		Excitability L_sup_front	VBM L_sup_front (*r=-0.18*)	NA	NA	0.039
		Excitability L_sup_pariet	VBM L_sup_pariet (*r=-0.19*)	NA	NA	0.028

		Excitability L_sup_temp	VBM L_sup_temp (*r=-0.21*)	NA	NA	0.017
		Excitability R_hippocampus	VBM R_hippocampus (*r=-0.19*)	NA	NA	0.031
		Excitability R_entorhinal	VBM R_entorhinal (*r=-0.20*)	NA	NA	0.019
		Excitability R_amygdala	VBM R_amygdala (*r=-0.33*)	NA	NA	< 0.001
		Excitability R_fusiform	VBM R_fusiform (*r=-0.21*)	NA	NA	0.018
		Excitability R_parahippoc	VBM R_parahippoc (*r=-0.20*)	NA	NA	0.033
		Excitability R_post_cing	VBM R_post_cing (*r=-0.20*)	NA	NA	0.024
		Excitability R_sup_temp	VBM R_sup_temp (*r=-0.22*)	NA	NA	0.010
		Excitability R_transv_temp	VBM R_transv_temp (*r=-0.20*)	NA	NA	0.020
MMSE*	Linear regression model	NA	NA	4.43	123	< 0.001
MoCA*	Linear regression model	NA	NA	4.58	120	< 0.001

CU cognitively unimpaired; MCI mild cognitive impairment; AD Alzheimer's disease; inf_temp inferior temporal; mid_temp middle temporal; parahippoc parahippocampal; post_cing posterior cingulate; sup_front superior frontal; sup_pariet superior parietal; sup_temp superior temporal; transv_temp transverse temporal; ^ sex and age adjusted; * sex, age and education adjusted.

Supplementary Table 4. Multiple linear regression analysis investigating the pathological effects on neuronal activity as predictors of MMSE and MoCA scores in the *Aβ, tau, Aβ·tau, microglial activation* influence model

	β	95% CI of β	p
**MMSE scores**			
Intercept	27.136	[21.110 34.161]	< 0.001
$\theta^{A\beta}$	0.373	[-0.296 1.041]	0.272
θ^{Tau}	0.151	[-0.524 0.837]	0.659
**$\theta^{A\beta\cdot Tau}$**	**1.530**	**[0.856 2.205]**	**< 0.001**
$\theta^{microglial\ act.}$	45	[-1.1402 0.249]	0.201
Sex	-0.960	[-2.348 0.429]	0.174
Age	0.009	[-0.080 0.099]	0.834
Education	0.029	[-0.163 0.221]	0.762
**MoCA scores**			
	β	95% CI of β	p
Intercept	22.145	[11.994 32.296]	< 0.001
**$\theta^{A\beta}$**	**1.004**	**[0.081 1.927]**	**0.033**
θ^{Tau}	0.129	[-0.791 1.048]	0.782
**$\theta^{A\beta\cdot Tau}$**	**1.966**	**[1.024 2.888]**	**< 0.001**
$\theta^{microglial\ act.}$	-0.609	[-1.565 0.347]	0.209
Sex	-1.624	[-3.570 0.322]	0.101
Age	0.058	[-0.073 0.189]	0.380
Education	-0.025	[-0.2920 0.2424]	0.855

The influences of Aβ plaques ($\theta_E^{A\beta}$), tau tangles (θ_E^{Tau}), the interaction of Aβ and tau ($\theta_E^{A\beta\cdot Tau}$) and microglial activation ($\theta_E^{microglial\ act.}$) on neuronal activity, sex, age and education were considered as predictors. Reported values are obtained coefficients (β), the 95% confidence intervals and the p-values for the t-statistic of the two-sided hypothesis tests. Significant terms (5% level) other than the intercepts are highlighted. MMSE: R^2=0.19, p < 0.001; MoCA: R^2=0.21, p < 0.001. MMSE, Mini-Mental State examination; MoCA, Montreal Cognitive Assessment.

Supplementary Table 5. Brain regions in the considered parcellation.

Hippocampus	Lateral Orbitofrontal[B]	Posterior Cingulate[B, r]
Entorhinal[B]	Medial Orbitofrontal[R]	Precentral
Amygdala	Lingual[L]	Precuneus[B, b]
Caudal Anterior Cingulate	Middle Temporal[B, l]	Rostral Anterior Cingulate
Caudal Middle Frontal[r]	Parahippocampal[B]	Rostral Middle Frontal[R]
Cuneus	Paracentral	Superior Frontal
Fusiform[B]	Pars Opercularis[L]	Superior Parietal[r]
Inferior Parietal[R, b]	Pars Orbitalis[B]	Superior Temporal
Inferior Temporal[B]	Pars Triangularis[B]	Supramarginal[L, b]
Isthmus Cingulate[L, b]	Pericalcarine[L]	Transverse Temporal
Lateral Occipital[B]	Postcentral	Insula

Regions with a superscript next to the name have statistically different microglial activation PET SUVRs [fALFF] when comparing the CU and AD groups: [B] [b] - bilaterally different, [L] [l] - only the values of the left hemisphere region is different, [R] [r] - only the values of the right hemisphere region is different. All regions had different amyloid-beta and tau PET SUVRs in comparisons of AD and CU subjects (ANCOVA post-hoc t-tests with age and sex as covariates).

Supplementary Table 6. Electrophysiological model parameters

Parameter	Definition	Value	Ref.
$\begin{bmatrix} E_0 \\ I_0 \end{bmatrix}$	Initial conditions	$\begin{bmatrix} 0.075 \\ 0.01 \end{bmatrix}$ (set to produce plausible simulated electrophysiological and BOLD signals)	See Supp. Fig 10.
τ_I	Time-constant controlling the decay of inhibitory activity after stimulation	0.02 s	[1]
τ_E	Time-constant controlling the decay of excitatory activity after stimulation	0.01 s	[1]
C_{II}	Local inhibitory-inhibitory connection strength	1.2	[2–4]
C_{EI}	Local excitatory-inhibitory connection strength	6	[2–4]
C_{EE}	Local excitatory-excitatory connection strength	6.4	[2–4]
C_{IE}	Local inhibitory-excitatory connection strength	4.8	[2–4]
P	Average constant external input received by the excitatory population	0.65 (set to produce plausible simulated electrophysiological and BOLD signals)	[2–4] Supp. Figure 10
a_I	Maximum slope of the inhibitory sigmoidal activation function	1	[1]
a_E	Maximum slope of the excitatory sigmoidal activation function	1	[1]
θ_I	Position of the inhibitory sigmoidal firing function' threshold for activation	4	[2–4]
θ_E	Position of the excitatory sigmoidal firing function' threshold for activation	Variable in [2.75,2.85] depending on the regional pathological loads<br><br>2.8 (in normal baseline conditions)	[1–5] (eq. 1)
η	Global coupling strength scaling the anatomical connectivity matrix C_{lk}	2 (set to produce plausible simulated electrophysiological and BOLD signals)	[1–5] Supp. Figure 10
N	Number of brain regions of interest	66	[6]
	Transient time	20 s	[7,8]

Supplementary Table 7. Metabolic/hemodynamic model parameters

Parameter	Definition	Value	Ref.
$\begin{bmatrix} g_{E0} \\ z_{E0} \\ g_{I0} \\ z_{I0} \\ f_0 \\ y_0 \\ b_0 \\ q_0 \end{bmatrix}$	Initial conditions	$\begin{bmatrix} 1 \\ 0 \\ 1 \\ 0 \\ 1 \\ 0 \\ 1 \\ 1 \end{bmatrix}$	7,9–11
h_E	Efficacy of glucose consumption response to excitation	1	7,9–11
h_I	Efficacy of glucose consumption response to inhibition	1	7,9–11
$\varkappa_E$	Time-constant of the excitatory glucose consumption impulse response.	1 s	7,9–11
$\varkappa_I$	Time-constant of the inhibitory glucose consumption impulse response.	1 s	7,9–11
c	Steepness of the sigmoid function x	2.5	7,9–11
d	Position of the threshold of the sigmoid function x	1.6	7,9–11
γ	Baseline ratio of excitatory to inhibitory synaptic activity in the voxel	5	7,9–11
x_0	Fraction of glucose following the glycogenolitic pathway at rest	$\frac{1}{1 + exp[c(d - 1(t))]}$	7,9–11
μ	Efficacy of blood flow response to excitation	0.8	7,9–11
$\varkappa_f$	Time constant for CBF response	1.7	7,9–11
$\varkappa_0$	Transit time through the balloon	1	7,9–11
ζ	Coefficient of the steady state flow-volume relationship	0.4	7,9–11
V_0	Baseline blood volume	0.03	7,9–11
Υ_0	frequency offset of a fully deoxygenated blood vessel at 3 T	80.6 s^{-1} (at 3 T)	12–14
r_0	Slope defining the dependence of the R2*relaxation rate on blood oxygenation	178 s^{-1} (at 3 T)	12–14
E_0	Baseline oxygen extraction fraction	0.4	12–14
ε	Intrinsic ratio of blood to tissue signals at rest	0.24	12–14
TE	Echo time	32.0 ms	https://triad.tnl-mcgill.com/

Supplementary Note 1

```
// program to calculate the personalized combined neuronal activity influences by Aβ, tau and
// Aβ · Tau

{       // definitions
Define surrogate optimization parameters (bounds and constraints, number of iterations)
Load the subject's Aβ, tau and fALFF (rs-fMRI) and anatomical connectivity matrix
Define the neuronal activity influence model (Eq. 1)
Define neural mass model and transformations to simulate the resting-state BOLD signal
Define the objective function (minimize correlation distance between real and simulated BOLD)
}

{       // optimization
FOR i = 1 TO 20       // different random optimization evaluation trials
        Perform surrogate optimization until the algorithm converges
                // At each iteration:
                // simulate the BOLD signal,
                // calculate similarity with the subject's real signal,
                // retain the best evaluation thus far
                // (performed by Matlab's surrogateopt.m)
        Save the optimized neuronal activity affectation parameters and optimization outputs
ENDFOR
}

{       // post-processing
Retain the optimization outcome with the lowest overall cost
Reconstruct hidden quantities of interest, e.g., neuronal excitabilities, spectral power, etc
}
```

Supplementary References

1. Abeysuriya, R. G. *et al.* A biophysical model of dynamic balancing of excitation and inhibition in fast oscillatory large-scale networks. (2018).

2. Gjorgjieva, J., Evers, J. F. & Eglen, S. J. Homeostatic activity-dependent tuning of recurrent networks for robust propagation of activity. *Journal of Neuroscience* **36**, 3722–3734 (2016).

3. Meijer, H. G. E. *et al.* Modeling focal epileptic activity in the Wilson-cowan model with depolarization block. *J Math Neurosci* **5**, 7 (2015).

4. Wilson, H. R. & Cowan, J. D. Excitatory and inhibitory interactions in localized populations of model neurons. *Biophys J* **12**, 1–24 (1972).

5. Daffertshofer, A. & van Wijk, B. C. M. On the Influence of Amplitude on the Connectivity between Phases. *Front Neuroinform* **5**, 6 (2011).

6. Klein, A. & Tourville, J. 101 Labeled Brain Images and a Consistent Human Cortical Labeling Protocol. *Front Neurosci* **6**, 1–12 (2012).

7. Sotero, R. C. & Trujillo-Barreto, N. J. Biophysical model for integrating neuronal activity, EEG, fMRI and metabolism. *Neuroimage* **39**, 290–309 (2008).

8. Stefanovski, L. *et al.* Linking Molecular Pathways and Large-Scale Computational Modeling to Assess Candidate Disease Mechanisms and Pharmacodynamics in Alzheimer’s Disease. *Front Comput Neurosci* **13**, 1–27 (2019).

9. Sotero, R. C. & Trujillo-Barreto, N. J. Modelling the role of excitatory and inhibitory neuronal activity in the generation of the BOLD signal. *Neuroimage* **35**, 149–165 (2007).

10. Sotero, R. C., Trujillo-Barreto, N. J., Jiménez, J. C., Carbonell, F. & Rodríguez-Rojas, R. Identification and comparison of stochastic metabolic/hemodynamic models (sMHM) for the generation of the BOLD signal. *J Comput Neurosci* **26**, 251–69 (2009).

11. Valdes-Sosa, P. A. *et al.* Model driven EEG/fMRI fusion of brain oscillations. *Hum Brain Mapp* **30**, 2701–2721 (2009).

12. Obata, T. *et al.* Discrepancies between BOLD and flow dynamics in primary and supplementary motor areas : application of the balloon model to the interpretation of BOLD transients. **21**, 144–153 (2004).

13. Simon, A. B. & Buxton, R. B. Understanding the dynamic relationship between cerebral blood flow and the BOLD signal: Implications for quantitative functional MRI. *Neuroimage* **116**, 158–167 (2015).

14. Archila-Meléndez, M. E., Sorg, C. & Preibisch, C. Modeling the impact of neurovascular coupling impairments on BOLD-based functional connectivity at rest. *Neuroimage* **218**, (2020).
