# Supplementary material for: Personalized whole-brain neural mass models reveal combined Aβ and tau hyperexcitable influences in Alzheimer’s disease
Source: Commun Biol. 2024 May 4;7:528. doi: 10.1038/s42003-024-06217-2 (PMC11069569; doi:10.1038/s42003-024-06217-2)
Supplement: Supplementary file 5 — Reporting Summary [file 42003_2024_6217_MOESM5_ESM.pdf]

nature portfolio

Corresponding author(s): Yasser Iturria-Medina

Last updated by author(s): Apr 4, 2024

Reporting Summary

Nature Portfolio wishes to improve the reproducibility of the work that we publish. This form provides structure for consistency and transparency in reporting. For further information on Nature Portfolio policies, see our Editorial Policies and the Editorial Policy Checklist.

Statistics

For all statistical analyses, confirm that the following items are present in the figure legend, table legend, main text, or Methods section.

n/a	Confirmed	
☐	☒	The exact sample size (*n*) for each experimental group/condition, given as a discrete number and unit of measurement
☐	☒	A statement on whether measurements were taken from distinct samples or whether the same sample was measured repeatedly
☐	☒	The statistical test(s) used AND whether they are one- or two-sided<br>*Only common tests should be described solely by name; describe more complex techniques in the Methods section.*
☐	☒	A description of all covariates tested
☐	☒	A description of any assumptions or corrections, such as tests of normality and adjustment for multiple comparisons
☐	☒	A full description of the statistical parameters including central tendency (e.g. means) or other basic estimates (e.g. regression coefficient) AND variation (e.g. standard deviation) or associated estimates of uncertainty (e.g. confidence intervals)
☐	☒	For null hypothesis testing, the test statistic (e.g. *F*, *t*, *r*) with confidence intervals, effect sizes, degrees of freedom and *P* value noted<br>*Give P values as exact values whenever suitable.*
☒	☐	For Bayesian analysis, information on the choice of priors and Markov chain Monte Carlo settings
☒	☐	For hierarchical and complex designs, identification of the appropriate level for tests and full reporting of outcomes
☐	☒	Estimates of effect sizes (e.g. Cohen's *d*, Pearson's *r*), indicating how they were calculated

Our web collection on statistics for biologists contains articles on many of the points above.

Software and code

Policy information about availability of computer code

Data collection	No software was used for data collection
Data analysis	This research used custom code for the neuronal activity simulations and quantification of the pathological effects. The pseudocode is available in the Supplementary Material (Supplementary Note 1). The code is available to editors and reviewers.<br>The code and instructions will are freely available at the Neuroinformatics for Personalized Medicine lab's website (NeuroPM, https://www.neuropm-lab.com/publication-codes.html) and the Zenodo repository (https://doi.org/10.5281/zenodo.10909567)

For manuscripts utilizing custom algorithms or software that are central to the research but not yet described in published literature, software must be made available to editors and reviewers. We strongly encourage code deposition in a community repository (e.g. GitHub). See the Nature Portfolio guidelines for submitting code & software for further information.

Data

Policy information about availability of data

All manuscripts must include a data availability statement. This statement should provide the following information, where applicable:

- Accession codes, unique identifiers, or web links for publicly available datasets
- A description of any restrictions on data availability
- For clinical datasets or third party data, please ensure that the statement adheres to our policy

The main source data supporting the findings of this study are available by submitting a data share request via https://triad.tnl-mcgill.com/contact-us/. All the data

collected under the TRIAD cohort is governed by the policies set by the Research Ethics Board Office of the McGill University, Montreal and the Douglas Research Center, Verdun. Other data and sources are available from the corresponding author on reasonable request.

Research involving human participants, their data, or biological material

Policy information about studies with human participants or human data. See also policy information about sex, gender (identity/presentation), and sexual orientation and race, ethnicity and racism.

Reporting on sex and gender	Post-hoc analyses of the estimated individual pathophysiological quantities of interest were performed considering relevant covariates, including sex and age. Gender was not available in the TRIAD cohort, and subsequently not considered here. Please, see Methods, Statistical Analyses for further details.
Reporting on race, ethnicity, or other socially relevant groupings	Relevant covariates age, sex and education (yrs) were considered in the statistical analyses (Methods, Statistical Analyses) Group-differences in the pathophysiological quantities of interest (average intra-brain theta and alpha1 power, excitatory firing activity and excitability) were evaluated with ANCOVA post-hoc t-tests accounting for sex and age. The regression analyses taking MMSE and MoCA scores as response variables, and the obtained personalized models' influences of Aβ, tau and the Aβ·tau interaction as predictors were adjusted for sex, age and education
Population characteristics	The majority of subjects (61.36%) were cognitively unimpaired; the remaining subjects either had mild cognitive impairment or Alzheimer's Disease dementia. The mean age at baseline was 70.9 y.o. APOE4 frequency was 0.36. See Suppementary Table 1 for further details.
Recruitment	All subjects in this study were part of the Translational Biomarkers in Aging and Dementia (TRIAD) cohort, a longitudinal imaging and biofluid cohort study of aging and AD. Participants were recruited through advertisements in the community, newspaper advertisements, word of mouth, and referrals from the McGill Centre for Studies in Aging. Evaluations of participants included a review of their medical history and an interview with the participant and their study partner followed by a neurologic examination by a dementia specialist and a neuropsychological examination. The main potential bias is participants' willingness to participate in the study, which may result in cognitive and other aspects of the investigated cohort being different from individuals who are unwilling to participate, or are unaware of this possibility.
Ethics oversight	This study's protocol was approved by McGill University's Institutional Review Board. Informed written consent was obtained from all participants.

Note that full information on the approval of the study protocol must also be provided in the manuscript.

Field-specific reporting

Please select the one below that is the best fit for your research. If you are not sure, read the appropriate sections before making your selection.

☒ Life sciences ☐ Behavioural & social sciences ☐ Ecological, evolutionary & environmental sciences

For a reference copy of the document with all sections, see nature.com/documents/nr-reporting-summary-flat.pdf

Life sciences study design

All studies must disclose on these points even when the disclosure is negative.

Sample size	All participants that had the required data were included in this study. No specific sample size calculations were performed.
Data exclusions	Only participants with "cognitively unimpaired" (N=81), "mild cognitive impairment" (N=35), or "Alzheimer's disease" (N=16) clinical and pathophysiological diagnoses were considered. Participants had all structural (T1) MRI, resting-state fMRI, Aβ (18F-NAV4694)-, tau (18F-MK-6240)- and microglial activation (11C-PBR28)- PET scans
Replication	Observations were consistent across non-overlapping groups of subjects representing different stages of the disease; cohorts other than TRIAD were not included in this study.
Randomization	No randomized trials were performed. The corresponding analyses were performed at the individual level. Diagnostic assignments were performed by clinical evaluation.
Blinding	Blinding was not relevant to our study. All our analyses were unsupervised, i.e. not requiring any a priori training or fitting on cognitive or behavioural variables.

Reporting for specific materials, systems and methods

We require information from authors about some types of materials, experimental systems and methods used in many studies. Here, indicate whether each material, system or method listed is relevant to your study. If you are not sure if a list item applies to your research, read the appropriate section before selecting a response.

Materials & experimental systems

n/a	Involved in the study
☒	☐ Antibodies
☒	☐ Eukaryotic cell lines
☒	☐ Palaeontology and archaeology
☒	☐ Animals and other organisms
☒	☐ Clinical data
☒	☐ Dual use research of concern
☒	☐ Plants

Methods

n/a	Involved in the study
☒	☐ ChIP-seq
☒	☐ Flow cytometry
☐	☒ MRI-based neuroimaging

Magnetic resonance imaging

Experimental design

Design type: Structural (T1) MRI, resting-state fMRI

Design specifications: n/a

Behavioral performance measures: n/a

Acquisition

Imaging type(s): Structural, resting-state functional

Field strength: 3T

Sequence & imaging parameters: T1 space sequence was performed in sagittal plane in 1 mm isotropic resolution; TE 2.96 ms, TR 2300 ms, slice thickness 1 mm, flip angle 9 deg, FOV read 256 mm, 192 slices per slab.
The resting-state fMRI acquisition parameters were: Siemens Magnetom Prisma, echo planar imaging, 860 time points, TR = 681 ms, TE = 32.0 ms, flip angle = 50 deg, number of slices = 54, slice thickness = 2.5 mm, spatial resolution = 2.5×2.5×2.5 mm3, EPI factor = 88.

Area of acquisition: Whole brain

Diffusion MRI: ☒ Used ☐ Not used

Parameters: 46 separate images, with 5 b0 images (no diffusion sensitization) and 41 diffusion-weighted images (b = 1000 s/mm2) from N = 128 subjects in the Alzheimer's Disease Neuroimaging Initiative (ADNI) (adni.loni.usc.edu)

Preprocessing

Preprocessing software: SPM12 (www.fil.ion.ucl.ac.uk/spm).

Normalization: All structural T1 images underwent non-uniformity correction using the N3 algorithm (Sled et al., 1998). Next, they were segmented into grey matter, white matter and cerebrospinal fluid (CSF) probabilistic maps, using SPM12 (www.fil.ion.ucl.ac.uk/spm). Grey matter segmentations were standardized to MNI space (Evans et al., 1994) using the DARTEL tool (Ashburner, 2007).
All other imaging modalities were registered to the native T1 and from there normalized at the MNI space by applying the T1's normalization transformation parameters. For further details, please see Methods.

Normalization template: ICBM152(MNI)

Noise and artifact removal: Motion correction across scans was performed in the resting state fMRI, using SPM12 (www.fil.ion.ucl.ac.uk/spm).

Volume censoring: n/a

Statistical modeling & inference

Model type and settings: n/a

Effect(s) tested: n/a

Specify type of analysis: ☐ Whole brain ☒ ROI-based ☐ Both

Anatomical location(s): 66 anatomically-defined regions covering all the brain’s grey matter, proposed by Klein A, Tourville J. 101 Labeled Brain Images and a Consistent Human Cortical Labeling Protocol. Front. Neurosci. 2012; 6: 171.

Statistic type for inference (See Eklund et al. 2016)	n/a
Correction	n/a

Models & analysis

n/a	Involved in the study
☒	☐ Functional and/or effective connectivity
☒	☐ Graph analysis
☒	☐ Multivariate modeling or predictive analysis
